# Analysis of tear fluid proteins: use of multiplex assays in profiling biomarkers of dry eye disease

**DOI:** 10.1186/1878-5085-5-S1-A129

**Published:** 2014-02-11

**Authors:** Suzanne Hagan, Alan Tomlinson, Louise Madden, Anne M Clark, Katherine Oliver

**Affiliations:** 1 Vision Sciences, Glasgow Caledonian University, Glasgow, UK; 2 Biological and Biomedical Sciences, Glasgow Caledonian University, Glasgow, UK

## Aims

Dry eye disease (DED) is a distressing condition that is commonly associated with ageing, contact lens wear and autoimmune disease. DED is one of the fastest-growing eye disorders in older populations (over-50s) and is significantly underdiagnosed. Multiplex studies of tear fluids from DED patient have suggested a role for inflammatory cytokines in its pathology. In this study, we investigated tear fluids from DED and normal subjects for a panel of 7 cytokines, using the multiplex bead assay.

## Methods

The study was composed of 15 DED subjects (3 males, 12 females) and 20 healthy controls (2 males, 18 females). All subjects provided informed consent and the study adhered to the Declaration of Helsinki tenets. Tear samples (1ml) were collected from the external canthus of eyes, using glass microcapillary tubes. Samples were diluted 1:50 and tears underwent analysis using a 7-plex bead assay (R and D Systems, UK). Cytokines were quantified using a Luminex IS200. Standard curves of known cytokine concentrations were used to calculate protein concentrations and data underwent analysis by an in-house statistician.

## Results

Detectable levels of IL-8 (> 4.05pg/ml) were observed in 12/15 DED subjects (mean 1156pg/ml) and 16/19 normals (mean 457.7pg/ml). Some variation in IL-8 levels was noted in DED subjects (246-4,391.5 pg/ml), but an overall trend for increased IL-8 was observed, compared to normals. Moreover, detectable levels of all 7 inflammatory proteins (IL1β, IL2, IL6, IL8, IL17, TNF-α and IFN-γ) were observed in 2 DED subjects, a result not observed in normals.

## Conclusions

An increased IL-8 level in DED patients suggests a function in ocular surface inflammation. This data confirms previous Multiplex bead studies, which have indicated a role for this protein in dry eye. Further studies may also shed light on roles for the other 6 cytokines detected in 2 DED subjects. Additionally, it appears that this technology is sensitive enough to detect low abundance cytokines in minute tear samples, and thus may be of future use as a tool for identifying potential biomarkers of DED.

